# Circulating programmed death ligand-1 (cPD-L1) in non-small-cell lung cancer (NSCLC)

**DOI:** 10.18632/oncotarget.24785

**Published:** 2018-04-03

**Authors:** Silvia Vecchiarelli, Francesco Passiglia, Armida D’Incecco, Marianna Gallo, Antonella De Luca, Elisa Rossi, Federica D’Incà, Gabriele Minuti, Lorenza Landi, Chiara Bennati, Michela Spreafico, Manolo D’Arcangelo, Valentina Mazza, Nicola Normanno, Federico Cappuzzo

**Affiliations:** ^1^ Department of Oncology and Hematology, AUSL della Romagna, Ravenna, Italy; ^2^ Department of Surgical, Oncological and Stomatological Disciplines, University of Palermo, Palermo, Italy; ^3^ Medical Oncology and Immunotherapy, University Hospital of Siena, Siena, Italy; ^4^ Cell Biology and Biotherapy Unit, Istituto Nazionale Tumori "Fondazione G Pascale"-IRCCS, Naples, Italy; ^5^ Fondazione Ricerca Traslazionale, Rome, Italy

**Keywords:** PD-L1, immunotherapy, biomarkers, non-small-cell lung cancer

## Abstract

**Background:**

This study aimed at investigating feasibility of programmed death ligand-1 (PD-L1) testing in plasma samples of advanced NSCLC patients receiving first-line treatment, assessing whether circulating (c)PD-L1 levels were modified by the therapy and whether baseline cPD-L1 levels were associated with patients’ clinical responses and survival outcome.

**Methods:**

Peripheral blood samples were collected from 16 healthy volunteers and 56 newly diagnosed NSCLC patients before and at 12th week during the course of first-line therapy. The level of PD-L1 was measured in plasma samples using the human (PD-L1/CD274) ELISA kit (CUSABIO, MD, USA). The Mann Whitney test or Fisher’s test were used for comparisons. Survival analysis was performed using Kaplan Meyer method, providing median and *p*-value.

**Results:**

Baseline median cPD-L1 was 42.21 pg/ml (range 12.00-143.49) in NSCLC patients and 37.81 pg/ml (range 9.73-90.21) in healthy control cohort (*p* = 0.78). Median cPD-L1 increased in patients treated with first-line chemotherapy (63.20 pg/ml vs 39.34 pg/ml; *p* = 0.002), with no changes in patients exposed to non-chemotherapy drugs (42.39 pg/ml vs 50.67 pg/ml; *p* = 0.398). Time to progression and overall survival were 4.4 vs 6.9 months (*p* = 0.062) and 8.8 vs 9.3 months (*p* = 0.216) in cPD-L1 positive vs cPD-L1 negative patients. Baseline cPD-L1 levels increased with the ascending number of metastatic sites, even if the association was not statistically significant (*p* = 0.063).

**Conclusions:**

This study showed that cPD-L1 testing is feasible, with chemotherapy influencing PD-L1 plasma levels. The possibility of using such test for predicting or monitoring the effect of immunotherapy or combination of chemotherapy and immunotherapy warrant further investigations.

## INTRODUCTION

A deeper understanding of the molecular basis of tumor immunogenicity and cancer immune-escape favored the development of a new class of drugs which are able to modulate the anti-tumor immune response [1, 2], ultimately leading to an impressive and durable clinical benefit in a significant subgroup of patients with advanced non-small cell lung cancer (NSCLC). Particularly to date we have three monoclonal antibodies (MoAbs), Nivolumab, Pembrolizumab and Atezolizumab, targeting the programmed cell death 1 (PD1)/programmed cell death 1 ligand (PD-L1) immune-checkpoint, approved by regulatory authorities for the treatment of advanced NSCLC [3]. Overall, four phase III randomized studies demonstrated that PD1/PD-L1 inhibitors are more effective and better tolerated than the second-line single agent chemotherapy [4–7], thus representing the new standard of care for NSCLC patients who experienced progression after platinum-combinations. A recent survival update of the CheckMate-003 phase I study revealed that about 15% of NSCLC patients were still alive after 5 years of therapy with nivolumab [8], thus suggesting that these drugs could offer the potential for a durable disease control and long-term survival in a subset of patients. Conversely, about 50% of pre-treated patients do not gain any benefit from immunetherapy [4–7], and a small subgroup of them develop “hyperprogression” or early death within the first 3 months of therapy with checkpoint inhibitors (ICIs) [9–11], making the identification of predictive biomarkers an urgent challenge for translational lung cancer research. The majority of studies including pre-treated NSCLC patients showed that the benefit of ICIs increased accordingly to the tumor PDL1-expression. However patients with PDL1 negative tumors also benefited from checkpoint inhibitors as compared to standard chemotherapy with docetaxel [4–7], suggesting that, because of its low sensitivity and specificity, PDL1 status alone may not be considered as an appropriate biomarker to exclude pre-treated patients from immunotherapy. Recently, Pembrolizumab revealed a significant superiority over platinum based chemotherapy as first-line treatment of non-oncogene addicted NSCLC patients whose tumors overexpressed PD-L1>50% [12], becoming the new backbone in this subgroup of patients who represent about 30% of the overall untreated population. In addition, recent studies showed that ICIs are effective in first-line setting irrespective of PD-L1 expression when used in combination with chemotherapy [13]. In light of these evidences, the PD-L1 testing has been incorporated within the international guidelines and it is now recommended together with the molecular testing for all patients with newly diagnosed advanced NSCLC, in order to ensure the most effective upfront treatment for each patient [3]. Even if limited by a lack of standardization in testing methods, the PD-L1 assessment by immune-histochemistry (IHC) on tumor tissue represents the current gold standard. However it’s not applicable in those patients whose tissue is not available at the time of diagnosis or tissue analysis results are not evaluable. Furthermore a biopsy sample is just a snapshot of the tumor not reflecting the overall microenvironment, and thus subjected to the intra-tumor heterogeneity. In addition, the immune response is a very complex and dynamic process taking place in different sites other than tumor-microenvironment, suggesting that the PD-L1 evaluation on tumor site could be not completely representative of the overall individual immune status. In the last decade an alternative not invasive approach, known as liquid biopsy, has been proposed to overcome all the aforementioned issues [14]. Epidermal growth factor receptor (EGFR) mutational testing by circulating tumor (ct) DNA analysis demonstrated an adequate diagnostic accuracy [15–18] and has been recently incorporated in the clinical management of all EGFR-mutated NSCLC patients who progressed after first-generation EGFR tyrosine kinase inhibitors (TKIs) and in a subgroup of patients with newly diagnosed metastatic disease who cannot undergo tumor biopsy or received uninformative results from tissue molecular analysis [3]. Even if the modulation of immune-system is a more complex and highly regulated process, however the identification and validation of potential biomarkers in the blood of patients could offer a valid tool in the hands of oncologists to easily monitor the efficacy of ICIs therapy. In the current study we aim to evaluate the feasibility of PD-L1 testing in the plasma of advanced NSCLC patients and to assess if circulating (c) PD-L1 levels may be modified by first-line therapies and are correlated with patients’ outcomes.

## RESULTS

### Patients’ characteristics and cPD-L1 detection

**Figure 1:**
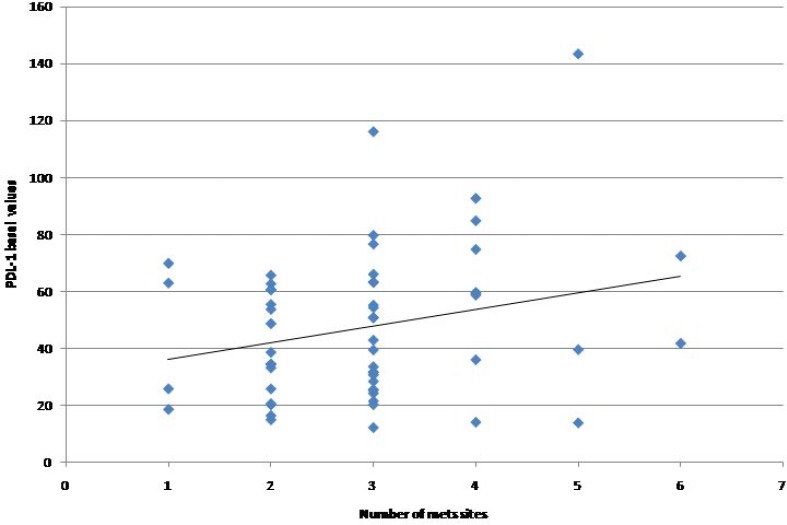
Baseline PD-L1 plasma levels according to the number of metastatic sites

From January 2013 to November 2015 a total of 56 patients with histologically or citologically confirmed diagnosis of advanced NSCLC who were candidate to receive first-line therapy and 16 healthy volunteers were included in the study. Median age was 70 years (range 48-85) and the majority were males and exhibited an Eastern Cooperative Oncology Group (ECOG) performance status score of 0 as reported in Table 1. The majority of patients were former smokers (57.1%) with adenocarcinoma histology (78.6%). Molecular alterations including *KRAS* mutations, *EGFR* mutations, and *ALK/ROS1* re-arrangements were detected in 23.2%, 19.6%, and 5.4% of analyzed tumor samples, respectively. Chemotherapy represented the first line treatment in 41 out of 56 (73.2%) patients, while 12 (21.4%) patients received targeted therapies including EGFR-TKIs (gefitinib *N* = 4, erlotinib *N* = 3, dacomitinib *N* = 2) and ALK/ROS1-TKIs (crizotinib, *N* = 3) and only 3 (5.4%) patients received immunotherapy with nivolumab within a clinical trial. Median PD-L1 plasma level at baseline was 42.21 pg/ml (range 12.00-143.49) in NSCLC patients, thus not significantly higher than that observed in the healthy control cohort (37.81 pg/ml, range 9.73-90.21; *p* = 0.78). Considering as cut-off value the median PD-L1 plasma level of 37.81 pg/ml detected in the healthy patients cohort, patients were classified as “cPD-L1 positive” (median plasma PD-L1>37.81 pg/ml; *N* = 32, 57.1%) or “cPD-L1 negative (median plasma PDL1<37.81 pg/ml; *N* = 24, 42.9%). As reported in Table 2, no significant association was observed between cPD-L1 levels and patients’ characteristics. A trend toward an increase of cPD-L1 according to the number of metastatic sites has been reported, even if it was not statistically significant (*p* = 0.063, Figure 1).

**Table 1:** Patients’ characteristics

Characteristic	Total (*n*)	%
**Total number of patients**	56	100
**Median age (years–range)**	70	48–85
**Gender**		
Male	38	67.9
Female	18	32.1
**Histology**		
Adenocarcinoma	44	78.6
Squamous-cell carcinoma	6	10.7
Not specified	6	10.7
**Smoking history**		
Never	11	19.6
Former	32	57.1
Current	13	23.2
**Performance status**		
0	38	67.8
1	10	17.9
2	6	10.7
3	2	3.6
**Number of metastaticsites**		
1–2	20	35.7
3–4	31	55.4
≥5	5	8.9
**First line treatment**		
Chemotherapy	41	73.2
Targeted therapies	12	21.4
Immunotherapy	3	5.4
***EGFR* status**		
Mutated^a^	11	19.6
Wild type	37	66.1
Unknown	8	14.3
***KRAS* status**		
Mutated^b^	13	23.2
Wild type	25	44.7
Unknown	18	32.1
***ALK* status**		
Rearranged	2	3.6
Wild type	37	66.1
Unknown	17	30.3
***ROS1* status**		1.8
Rearranged	1	32.1
Wild type	18	66.1
Unknown	37	
**Triple negative**^c^	9	16.1

^**a**^EGFR mutations included: exon 18 = 1 (9.0%); exon 19 = 4 (36.4%); exon 20 = 2(18.2%); exon 21 = 4 (36.4%); ^**b**^KRAS mutations included: codon 12 = 13(100%); ^c^Triple negative included *EGFR/KRAS/ALK/ROS1* wild-type patients.

**Table 2:** Correlation between plasma PD-L1 levels and patients’ characteristics

Patients characteristic	PD-L1 classes (based on medianvalue) - *N* (%)		*p* value
	negative	positive	
**Age**			0.58
≤70.4	13 (46.4)	15 (53.6)	
>70.4	11 (39.3)	17 (60.7)	
**Gender**			0.86
Male	16 (42.1)	22 (57.9)	
Female	8 (44.4)	10 (55.6)	
**Smoking history**			0.71
Never/Former	19 (44.2)	24 (55.8)	
Current	5 (38.5)	8 (61.5)	
**Number of metastaticsites**			0.06
1	2 (50.0)	2 (50.0)	
2	8 (50.0)	8 (50.0)	
3	11 (47.8)	12 (52.2)	
4	2 (25.0)	6 (75.0)	
5	1 (33.3)	2 (66.7)	
6	0 (0.0)	2 (100.0)	
***EGFR* status**			0.97
Mutated^a^	5 (45.5)	6 (54.5)	
Wild type	17 (45.9)	20 (54.1)	
***KRAS* status**			0.42
Mutated^b^	5 (38.5)	8 (61.5)	
Wild type	13 (52.0)	12 (48.0)	
***ALK* status**			0.79
Rearranged	1 (50.0)	1 (50.0)	
Wild type	15 (40.5)	22 (59.5)	
***ROS1* status**			0.36
Rearranged	1 (100.0)	0 (0.0)	
Wild type	6 (33.3)	12 (66.7)	

^**a**^EGFR mutations included: exon 18 = 1 (9.0%); exon 19 = 4 (36.4%); exon 20 = 2 (18.2%); exon 21 = 4 (36.4%); ^**b**^KRAS mutations included: codon 12 = 13 (100%).

### Effect of treatments on cPD-L1 levels

**Figure 2:**
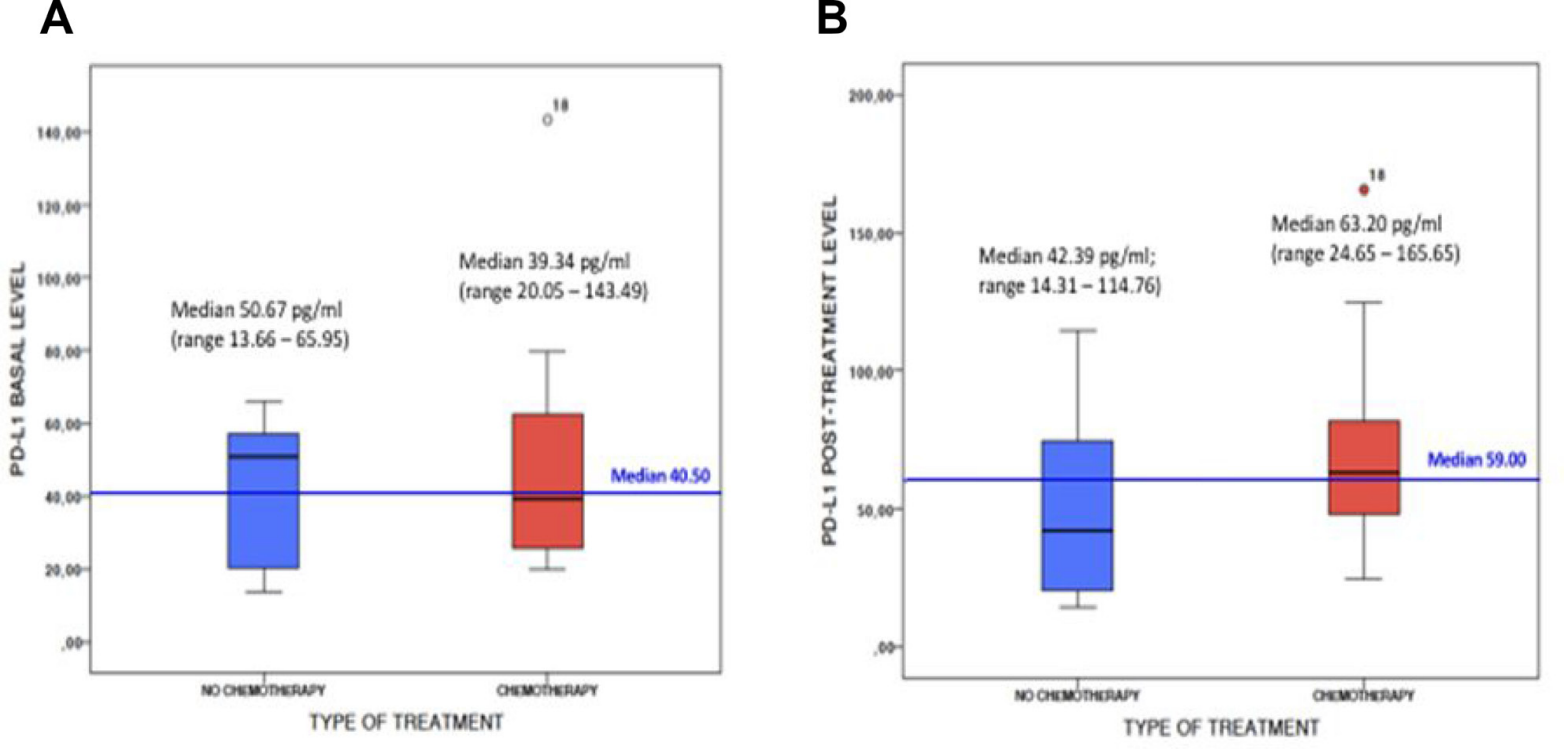
PD-L1 plasma levels at baseline (**A**) and after 3 months of first-line treatment (**B**) in subgroup of patients receiving chemotherapy (red) or no chemotherapy (blue) agents.

In 25 out of 56 patients evaluable after 3 months of first-line therapy, median PD-L1 plasma levels significantly increased as compared to baseline median value (58.63 pg/mL, *p* = 0.04). Among 18/41 patients treated with first-line chemotherapy-regimens and evaluable after 3 months, we observed a significant increase of the median cPD-L1 (63.20 pg/ml (range 24.65 – 165.65) versus 39.34 pg/ml (range 20.05 – 143.49), *p* = 0.002). Conversely, among 7/12 patients treated with no-chemotherapy agents (dacomitinib *N* = 2, gefitinib *N* = 2, erlotinib *N* = 1, nivolumab *N* = 2) and evaluable after 3 months, not significant changes in median cPD-L1 has been observed (42.39 pg/ml (range 14.31 – 114.76) vs 50.67 pg/ml (range 13.66 – 65.95), *p* = 0.398) (Figure 2).

### cPD-L1 level and tumor response

After 3 months of first-line treatment, 47 of 56 (83.9%) NSCLC patients were evaluable for tumor response. Among them, one patient (2.1%) experienced complete response (CR), 20 (42.5%) had a partial response (PR), 19 (40.4%) stable disease (SD), and 7 (14.9%) progression disease (PD). No significant differences in overall response rates (ORR: CR+PR) have been observed between cPDL1 positive and cPDL1 negative patients in the overall NSCLC population (ORR: 43.5% vs 45.8%; *p* = 0.87), in patients treated with chemotherapy-regimens (ORR: 47.1% vs 40%, *p* = 0.735), and in patients treated with no chemotherapy regimens (ORR: 33.3% vs 55.6%; *p* = 0.608), as illustrated in Table 3.

**Table 3:** Tumor response rate in NSCLC patients

Type of treatment	PD-L1 status	ORR-*N* (%)		*p*-value
		CR + PR	SD + PD	
**All treatment**	PD-L1 positive	10 (43.5)	13 (56.5)	0.87
	PD-L1 negative	11 (45.8)	13 (54.2)	
**Chemotherapy**	PD-L1 positive	8 (47.1)	9 (52.9)	0.73
	PD-L1 negative	6 (40.0)	9 (60.0)	
**No chemotherapy**	PD-L1 positive	2 (33.3)	4 (66.7)	0.60
	PD-L1 negative	5 (55.6)	4 (44.4)	

ORR = overall response rate; CR = complete response; PR = partial response; SD = stable disease; PD = progression disease.

### cPD-L1 level and patients’ survival outcomes

**Figure 3:**
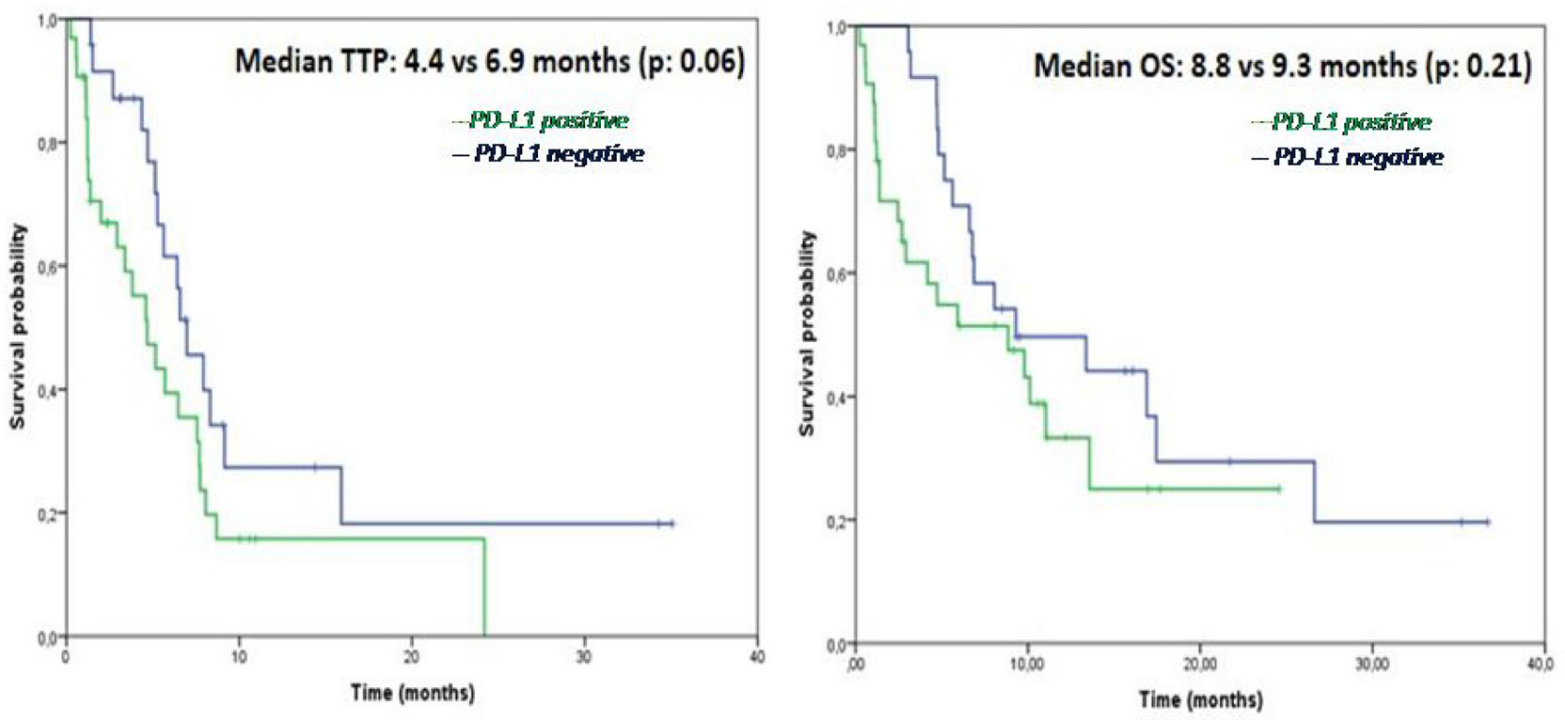
Kaplan-Meier analysis of time to progression (TTP) (**A**) and overall survival (OS) (**B**) in NSCLC patients according to the baseline cPD-L1 status.

At a median follow-up time of 14.7 months (range 2–36), disease progression occurred in 41 patients, while 36 patients died because of tumor progression, and 20 patients were still alive at the time of data analysis. Median time to progression (TTP) was 5.6 months and median overall survival (OS) was 8.8 months in the overall NSCLC population. As showed in Figure 3, no difference in TTP nor in OS were observed in cPD-L1 positive as compared to cPD-L1 negative patients (TTP: 4.4 versus 6.9 months, *p* = 0.062; OS: 8.8 versus 9.3 months, *p* = 0.216). No differences in TTP nor in OS were observed between cPD-L1 positive and negative subgroups even when the analysis was restricted to patients treated with chemotherapy (TTP: 3.3 versus 5.6 months, *p* = 0.623; OS: 4.1 versus 6.8 months, *p* = 0.322) or no chemotherapy-regimens (TTP: 4.6 versus 15.9 months, *p* = 0.188; OS: 9.7 versus 17.4 months, *p* = 0.887).

## DISCUSSION

This proof-of concept study demonstrated the feasibility of PD-L1 testing in plasma samples of patients with advanced NSCLC, showing that the cPD-L1 expression levels may be significantly modified by the standard first-line chemotherapy. As tumor tissue PDL1 assessment by IHC is currently recommended by all the international guidelines as diagnostic test for patients with advanced NSCLC who are candidate to receive upfront treatment [3], the possibility to detect and monitor PDL1 expression in the plasma before and during the course of therapies could play an important role in the management of NSCLC patients, especially when tissue is not available at the time of diagnosis or tissue analysis results are not evaluable. Particularly, monitoring the cPDL1 level changes during first-line treatment revealed opposite trends in predefined subsets of patients. Indeed the results of our analysis showed that the median cPDL1 levels significantly increased (63.20 pg/ml vs 39.34 pg/ml, *p* = 0.002) in patients receiving first-line chemotherapy, providing a potential biological explanation to the efficacy of immunotherapy plus chemotherapy combinations even in the subgroup of patients with PD-L1 negative tumors at baseline IHC assessment [13]. Conversely in the small subgroup of patients treated with non-chemotherapy agents, largely represented by EGFR-mutated patients, the high median baseline PD-L1 value decreased (42.39 pg/ml vs 50.67 pg/ml, *p* = 0.398) during EGFR-TKI treatment. These data are in line with recent evidences showing that PD-L1 is constitutively expressed by EGFR-mutated tumors and is subjected to decrease after EGFR-TKIs [19–21], thus making these patients less responsiveness to subsequent anti-PD1 therapies. Furthermore we know that although the high level of PD-L1 expression, oncogene-addicted NSCLC are associated with a very low tumor mutational burden [22] and a non-inflamed tumor microenvironment, and are characterized by neither an immune response nor a T-cell tumor infiltration [19, 23], thus less likely to respond to immunotherapy [23, 24]. Our findings demonstrated that cPDL1 expression levels may be significantly modified by the patients’ anticancer treatments. As compared to the archival tumor biopsy, the longitudinal monitoring of cPDL1 levels in the plasma of NSCLC patients would allow to easily identify any significant variations during the course of therapies which can inform oncologists about treatment decisions in everyday practice. Furthermore several evidences revealed that PDL1 is an heterogeneous and dynamic biomarkers, subjected to both space and time variability [25]. Thus a single tumor biopsy could not reflect neither the overall tumor microenvironment nor the systemic immune response in different individuals [26]. In this regards the evaluation of cPDL1 in the plasma of NSCLC patients could be more representative of the overall immune status at single patient level in a determinate time-point of the disease course supporting the oncologists in their clinical decisions. Pre-clinical data showed that only PD-L1 positive, but not PD-L1 negative cell lines secreted cPD-L1 in their supernatant [27], suggesting that the cPD-L1 could derive from the cell surface protein through different mechanisms including proteolytic cleavage or alternative mRNA splicing. However the source of cPD-L1 remains uncertain and these preliminary data need to be proven in clinical setting. In addition to that, the results of our study showed an interesting trend toward a not significant increase of cPD-L1 levels according to the number of metastatic sites in the included population. As reported in previous studies [28], this association could be the result of a greater release of tumor DNA into the blood of patients with high tumor burden, suggesting that the extension of the disease could significantly influence our ability to detect PD-L1 in the plasma of patients with advanced NSCLC. This has been already demonstrated for EGFR-mutations detection by ctDNA analysis [29–31] in EGFR-positive NSCLC patients but need to be further investigated for cPD-L1 and other potential biomarkers detected in the blood of NSCLC patients receiving ICIs. Finally some limiting factors of this study need to be mentioned, including the low number of evaluated patients, the short follow-up, the heterogeneity of both tumors’ molecular profile and treatment regimens, and the lack of a tumor tissue analysis as reference test because of the unavailability of sufficient archival tissue for PD-L1 expression assessment. Thus it remains still unknown the relationship between cPD-L1 levels and tumor tissue PD-L1 expression or other peripheral blood parameters associated with systemic inflammatory state such as neutrophil-lymphocytes ratio. It would be interesting also to monitor the cPDL-1 levels beyond 12 weeks in order to investigate any significant modifications in NSCLC patients who respond to immunotherapy or chemo-immunotherapy combinations.

In conclusion the results of this study demonstrated that PDL1 testing in the plasma of advanced NSCLC patients is feasible and cPDL1 levels significantly increase during first-line chemotherapy. These data suggest cPD-L1 as a potential biomarker in the early prediction and real-time monitoring of both immunotherapy and chemo-immune combinations efficacy, warranting further investigations in prospective clinical studies including larger cohort of patients and longer follow-up. A dynamic assessment of cPD-L1 in association with other emerging biomarkers could allow a better patients’ stratification favoring the development of personalized immune-treatment strategies.

## MATERIALS AND METHODS

### Patients

Patients were eligible if they had histologically or cytologically confirmed diagnosis of non- squamous or squamous NSCLC, stage IV (according to Version 8th of the International Association for the Study of Lung Cancer (IASLC) TNM Staging System), Eastern Cooperative Oncology Group (ECOG) performance-status score <3, and had not previously received any systemic treatment for advanced/metastatic disease.

A cohort of healthy volunteers (individuals who were not affected by oncological, autoimmune, metabolic and infectious diseases), was also included in the study for plasma PD-L1 assessment, and the results obtained were compared with those observed in NSCLC patients.

The study was conducted in accordance with the International Conference on Harmonization Guidelines on Good Clinical Practice and the Declaration of Helsinki.The trial protocol was previously approved by the local Independent Ethics Committee and both the cancer patients and the healthy volunteers provided a written informed consent before enrollment.

### Study design and treatment

From January 2013 to November 2015 eligible patients were included in thisprospective cohort study and received first-line standard systemic treatment according to their tumors’ histology and molecular profile. All the treatments were continued until disease progression or the occurrence of an unacceptable level of toxicity, or the completion of permitted cycles (up to 4-6 for platinum-based chemotherapies). Mainteinance treatment with single agent pemetrexed was allowed for patients with non-squamous NSCLC who had not progression after 4 cycles of chemotherapy with platinum-pemetrexed. Radiological evaluation of treatment efficacy by CT-scan was performed after 12 weeks of therapy and responses were evaluated by Response Evaluation Criteria in Solid Tumors (RECIST)version 1.1.

### Objectives of the Study

The main objective of the study was to evaluate the feasibility of PD-L1 testing in plasma samples of patients with advanced NSCLC and to determine how the expression levels of cPD-L1 might be modified by the standard first-line treatment.

Secondary objective of the study was to investigate the relationship between the plasma cPD-L1 levels of NSCLC patients, their clinical characteristics and treatment efficacy outcomes, including ORR, TTP and OS.

### Circulating PD-L1 assessment

Peripheral blood samples were collected frompatients included in the study before and at 12th weeks during the course of first-line therapy, according to a simple and standardized protocol and stored frozen as 500 ml aliquots at –80° C. The expression levels of PD-L1 were assessed in plasma samples using the Human programmed death ligand-1 (PD-L1/CD274) ELISA kit (CUSABIO, MD, USA). Plasma samples were centrifuged for 15 minutes at 1000 × g and the assay was performed according to the manufacturer’s instructions. Briefly, 96-well plates were incubated with standards and plasma samples for 2 hours at 37° C. Then, a biotin-conjugated antibody specific for PD-L1 was added to each well. After several aspiration/wash processes, horseradish peroxidase (HRP)-conjugated avidin was added. After incubation with a substrate solution, samples absorbance was read at 450 nm with the IMarkMicroplate Absorbance Reader (Biorad, Italy). The concentrations of PD-L1 were calculated according to standard curves.

### Statistical analysis

The median PD-L1 plasma levels were calculated in healthy volunteers, and in NSCLC patients before and after 12 weeks of therapy. The Mann Whitney test was used for intergroup comparisons of two independent samples while Fisher’s test was used for categorical values.

Paired Wilcoxon test was used to compare median PD-L1 before and after first line therapy. For efficacy analysis, patients were grouped according to their plasma PD-L1 concentration into “positive” if they had plasma PD-L1 concentration higher than median PD-L1 value observed in the healthy population or “negative” if they had plasma PD-L1 concentration below the median PD-L1 valueobserved in the healthy population. Patients’ clinical-pathological characteristics and associations with plasma PD-L1 level were examined with a descriptive analysis comparing the differences by *χ*^2^ test or Fisher’s exact test as appropriate. A *p*-value < 0.05 was considered significant.

Efficacy outcomes, including ORR, TTP and OS defined as the time between the date of inclusion and the date of disease progression or death, respectively, were assessed in cPDL1 positive vs cPDL1 negative patients both in the overall cancer population and in pre-defined subgroups of patients.

Survival analysis was performed using Kaplan Meyer method, providing median and *p*-values, with the use of the logrank test for comparisons. A *p*-value < 0.05 was used as threshold for statistical significance. All the statistical analysis were performed using SPSS Statisticssoftware version 20 (IBM, Armonk, New York, USA).

## Abbreviations

cPD-L1: circulating programmed cell death 1 ligand
CR: complete response
ct-DNA: circulating tumor DNA
ECOG: Eastern Cooperative Oncology Group
EGFR: Epidermal growth factor receptor
IASLC: International Association for the Study of Lung Cancer
ICIs: immune checkpoint inhibitors
IHC: immune-histochemistry
MoAbs: monoclonal antibodies
NSCLC: non-small cell lung cancer
ORR: overall response rates
PD: progression disease (PD)
PD1: programmed cell death 1
PD-L1: programmed cell death 1 ligand
PR: partial response
SD: stable disease
TKIs: tyrosine kinase inhibitors
